# Changes in BMI after treatment of testicular cancer are due to age and hormonal function and not chemotherapy

**DOI:** 10.1038/sj.bjc.6601178

**Published:** 2003-09-09

**Authors:** R A Huddart, A Norman

**Affiliations:** 1 Academic Unit of Radiotherapy & Oncology, Institute of Cancer Research and Royal Marsden Hospital, Downs Road, Sutton, Surrey SM2 5PT, UK; 2 Department of Computing & Information, Institute of Cancer Research and Royal Marsden Hospital, Downs Road, Sutton, Surrey SM2 5PT, UK

**Sir**,

We read with interest the article of Nord *et al* (2003) who reported excessive increases in body mass index (BMI) after chemotherapy in long-term survivors of testicular cancer. We believe this study presents important data but do not believe that the data presented fully support the authors’ conclusions. We have also been interested in the long-term effects of treatment on testicular cancer patients and have investigated a similar cohort of 680 patients, with at least one remaining testis, treated at our institution between 1982 and 1992. This consisted of 169 patients who had orchidectomy as the only treatment, 272 patients treated by chemotherapy, 158 treated by radiotherapy and 81 treated by both radiotherapy and chemotherapy. Full details of the study are reported elsewhere (Huddart *et al*, 2003). As part of this study, we investigated the relationship between BMI, clinical- and treatment-related parameters. Like Nord *et al*, patients who received chemotherapy demonstrated a clear increase in weight and resultant BMI with follow-up (*P*<0.001), with a mean annual increase of BMI of 0.264 kg m^−2^ year^−1^. Pretreatment BMI was not available for other groups so data on annual increase for these patients are not available.

On the basis of our data and those presented by Nord *et al*, we do not believe that treatment received has a direct significant effect on the long-term BMI. In support of this assertion is the lack of difference in the follow-up BMI according to treatment received in both our study (see Table 1

**Table 1:** Analysis of BMI in germ cell tumour patients treated at The Royal Marsden Hospital according to treatment group, age at last follow-up and hormonal status

		**Body mass index (kg/m^2^**)		
	***N***	**Median (IQR)**	**Mean (sd)**	**Significance^a^ (v baseline)**
*Treatment group*				
Chemotherapy	263	25.9	26.2	*P*=0.11
		(23.7–28.1)	(3.8)	
Radiotherapy	156	26.0	27.11	*P*=0.947
		(24.3–29.6)	(4.2)	
Chemotherapy/	78	26.9	26.7	*P*=0.998
Radiotherapy		(23.7–28.9)	(3.8)	
Surveillance	164	26.37	28.9	—
		(24.4–28.8)	(4.4)	
				
*Age at last follow-up*				
<30 years		24.4	26.1	
30–39 years		25.7	26.3	
40–49 years		26.1	26.6	
⩾50 years		26.7	27.1	*P* (for trend)=0.055
				
*Hormone levels* (testosterone)				
Normal	538	25.7		
		(23.6–28.1)		
Low	100	28.6		*P*<0.001
		(26.7–31.4)		

a Assessed by Mann–Whitney *U*-test for nonparametric variables. Note that these data are not normally distributed, so inclusion of mean levels is for comparative purposes with the data of Nord *et al* (2003).

) and in the study of Nord *et al* (Figure 2B in their paper). Nord *et al* has suggested that after chemotherapy, there is a higher annual increase of BMI in chemotherapy patients compared to controls. We do not have direct control data available on this issue, but we believe on review of the presented data that this assertion is flawed. If chemotherapy was to have an effect on annual BMI increase, we find it surprising that patients treated by orchidectomy only (and not orchidectomy and radiotherapy) also had a greater annual increase in BMI than controls. We believe a likely explanation for this discrepancy is revealed by careful examination of the age distribution of controls and treated patients. The average age of the control group (at last follow-up) is 8 years higher than the surgically treated and chemotherapy-treated patients (even greater at baseline). Our data (Table 1) and those of Nord *et al* (their Figures 1A and 2A) show a clear relationship between age and BMI; both at baseline and on follow-up. The data presented by Nord *et al* in Figure 3A confirm that the rate of increase of BMI is greatest in younger patients, with no obvious difference between controls and testicular cancer patients. We see a similar trend in our data. Thus, the greater change in BMI of chemotherapy-treated patients compared to controls is likely to be due to the greater rate of change in BMI between 30 and 44 year patients (age at baseline and follow-up of surgical and chemotherapy patients) compared to 41 and 52 years (age of control group). In support of this is the observation that patients treated with radiotherapy, who have an age at follow-up similar to controls, have a similar rate of increase of BMI to controls (Nord *et al*, Figure 1A).

This does not exclude important changes occurring in the BMI of testicular cancer survivors. However, we believe that these are more likely to be related to the underlying patient characteristics and the sequelae of orchidectomy. In our series of patients, we found that 100 out of 638 (15.7%) had low testosterone levels (⩽10 ng ml^−1^) at long-term follow-up. These patients had a significantly higher BMI compared to those with normal testosterone levels (median BMI of 28.6 compared to 25.7 kg m^−2^;*P*<0.001). In total, 88% of patients with low testosterone have a BMI in the overweight range (⩾25 kg m^−2^) compared to 58% of those with normal testosterone (Figure 2). When we undertook a linear regression analysis with age either at last follow-up or at treatment and testosterone status (normal *vs* low) at last follow-up, both significantly predicted for increased annual rate of change of BMI (both *P*<0.001). A model including age at last follow-up (which was a more robust predictor than age at treatment) and testosterone produced a model that predicted 18.6% of the variation observed. Although this is still a weak model, it is a stronger predictor of BMI increase than that produced by the Nord *et al* model.

The effect on survivors of raised BMI remains unclear. We have confirmed that there is a significant association between BMI and blood pressure in our series of testicular survivors (Table 2

**Table 2:** Association of overweight BMI with hormonal status and blood pressure

	**Body mass index**		***P*-value**
	**<25**	**⩾25**	
Testosterone status (% of patients)			
Normal	41%	58%	*P*<0.001^a^
Low	12%	88%	
			
Blood pressure (mmHg^−1^)			
Systolic (median)	130	140	*P*<0.001^b^
Diastolic (median)	80	90	*P*<0.001^b^

a *χ*^2^ test.

b Mann–Whitney *U*-test.

), but no association with cardiac events after a median of 10 years of follow-up (HR 1.01 95% CI 0.94–1.09; *P*=0.8) (Huddart *et al*, 2003).

In conclusion, in testicular cancer survivors, increases in BMI are associated with age and residual gonadal function, but the evidence of a direct treatment-related effect is weak at best. An increased BMI is associated with raised blood pressure but to date not with cardiac morbidity.
